# Supplementary material for: HDAC/σ1R Dual-Ligand as a Targeted Melanoma Therapeutic
Source: Pharmaceuticals (Basel). 2025 Jan 28;18(2):179. doi: 10.3390/ph18020179 (PMC11859726; doi:10.3390/ph18020179)
Supplement: Supplementary file 1 [file pharmaceuticals-18-00179-s001.zip › pharmaceuticals-3419154-supplementary.pdf]

Supporting Information

HDAC/σ1R Dual-Ligand as a Targeted Melanoma Therapeutic

Claudia Giovanna Leotta [1,2,†], Carla Barbaraci [3,4,†], Jole Fiorito [5,6], Alessandro Coco [3], Viviana di Giacomo [7], Emanuele Amata [3], Agostino Marrazzo [3,*], Giovanni Mario Pitari [1,2,*]

[1] Dream Factory Lab, Vera Salus Ricerca S.r.l., 96100 Siracusa, Italy
[2] J4Med Lab, Via Paolo Gaifami 9, 95126 Catania, Italy
[3] Department of Drug and Health Sciences, University of Catania, 95125 Catania, Italy
[4] Institut Català d'Investigació Química (ICIQ), Avinguda dels Països Catalans 16, 43007 Tarragona, Spain
[5] Department of Biological and Chemical Sciences, New York Institute of Technology, Old Westbury, NY 11568, USA
[6] Department of Medicine, Columbia University, New York, NY 10032, USA
[7] Department of Pharmacy, University "G. d'Annunzio", Chieti-Pescara, Via dei Vestini 31, 66100 Chieti, Italy
* Correspondence: marrazzo@unict.it (A.M.); giovanni.pitari@verasalusricerca.it (G.M.P.)
† These authors contributed equally to this work.

EXPERIMENTAL SECTION

Table of content

Inhibition of HDAC activity in HCT116, MCF7, PC3 and AGS cells by 5c (Figure S1)	S2
Regulation of total tube length, nodes, and mesh area in angiogenesis assays (Figure S2)	S3
Cell spreading counting (Table S1)	S4
NMR spectra of synthesized compounds (Figures S3-4)	S5-6
HRMS spectra of synthesized compounds (Figures S5-6)	S7-8

Inhibition of HDAC activity in HCT116, MCF7, PC3 and AGS cells by 5c

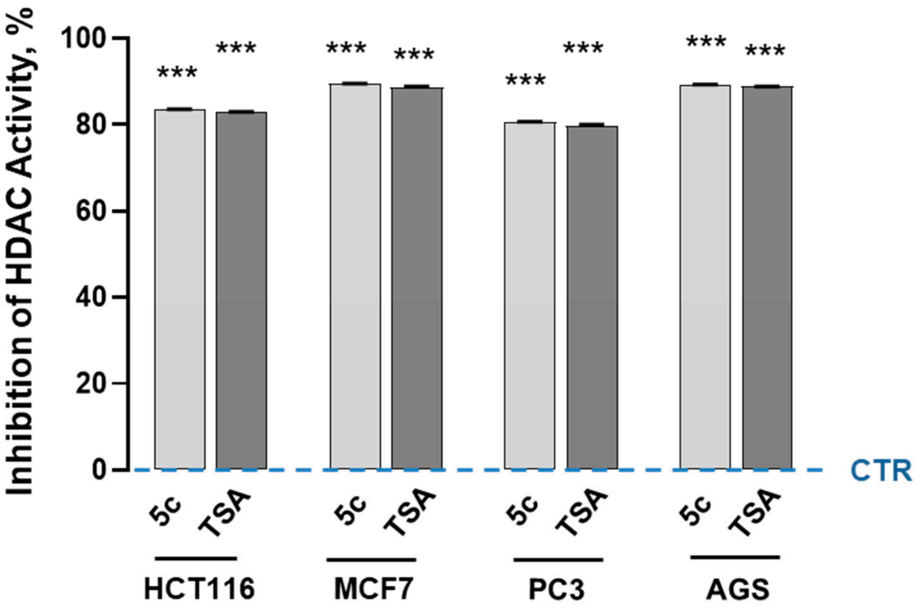

Figure S1. Inhibition of HDAC enzymatic activity in various human cancer cells by **5c**. HCT116, colon adenocarcinoma cells; MCF7, breast carcinoma cells; PC3, prostate adenocarcinoma cells; AGS, gastric adenocarcinoma cells. TSA, trichostatin A (positive control). HDAC enzymatic activity was measured as described in Materials and Methods. Results are percentages of inhibition of the vehicle DMSO control (CTR; indicated as a dashed blue line). ***, $p < 0.001$ *vs* CTR by Student's t-test.

Regulation of total tube length, nodes, and mesh area in angiogenesis assays

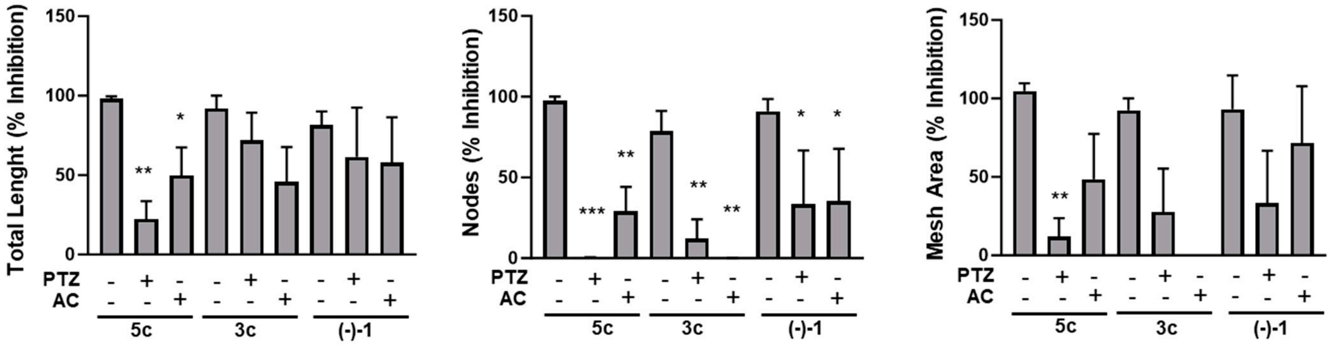

Figure S2. Inhibition of vascular-like structures in mouse endothelial C166 cells by **5c**, **3c**, and (-)-1. Capillary tube-like structures were stimulated with VEGF-A (80 ng/mL). Quantification of vascular networks were performed with the angiogenesis analyser tool of the NIH-Image-J software. Three parameters were measured: total length, number of nodes and total mesh area. Results were expressed as inhibition activity (%), calculated as: 100 – [(A/B) x 100], where A is the value observed in the presence of 5c or (-)-1 (alone or plus pentazocine/AC927) and B is the correspondent value observed in the VEGF-A condition. Treatments included 5 μM of **5c**, **3c** or (-)-1, alone or in the presence of pentazocine (PTZ, 2 μM) or AC927 (AC, 2 μM). *, p<0.05; **, p < 0.01 ; ***, p < 0.001 *vs.* respective testing compound alone by one-way ANOVA.

Cell spreading counting

Table S1. HDAC/σ_1R dual-ligand **5c**, hydroxamic-null precursor **3c** and positive control (-)-1 inhibited membrane protrusion formation in σ1R expressing UM 92-1 cells, but not in σ1R-deficient breast cancer MCF7 cells. Number of cells with both rounded and migratory (flattened cells, forming membrane protrusions such as lamellipodia and filopodia) morphology were quantified and expressed as percentage of total cell populations, as detailed in Materials and Methods. At least 400 cells per condition were quantified in each experiment.

Cell Line	Treatment	Cell Morphology	Exp 1 (%)	Exp 2 (%)	Exp 3 (%)
92-1	5c, 100 nM	Flat, with protrusions<br>Rounded	37,23<br>62,77	27,77<br>72,23	37,27<br>62,73
	5c, 5 µM	Flat, with protrusions<br>Rounded	28,48<br>71,52	17,22<br>82,78	33,05<br>66,95
	3c, 100 nM	Flat, with protrusions<br>Rounded	36,01<br>63,99	25,21<br>74,79	33,92<br>66,08
	3c, 5 µM	Flat, with protrusions<br>Rounded	45,38<br>54,62	18,28<br>81,72	39,44<br>60,56
	(-)-1, 100 nM	Flat, with protrusions<br>Rounded	33,84<br>66,16	21,33<br>78,67	34,97<br>65,03
	(-)-1, 5 µM	Flat, with protrusions<br>Rounded	19,39<br>86,70	13,49<br>86,51	26,09<br>73,91
	Vehicle (DMSO)	Flat, with protrusions<br>Rounded	47,31<br>52,69	43,46<br>56,54	42,74<br>57,26
MCF-7	5c, 100 nM	Flat, with protrusions<br>Rounded	47,13<br>52,87	44,67<br>55,33	39,07<br>60,93
	3c, 100 nM	Flat, with protrusions<br>Rounded	49,37<br>50,63	50,89<br>49,11	46,27<br>53,73
	(-)-1, 100 nM	Flat, with protrusions<br>Rounded	45,95<br>54,05	48,03<br>51,97	40,28<br>59,72
	Vehicle (DMSO)	Flat, with protrusions<br>Rounded	49,86<br>50,14	55,17<br>44,83	44,63<br>55,37

NMR spectra of synthesized compounds

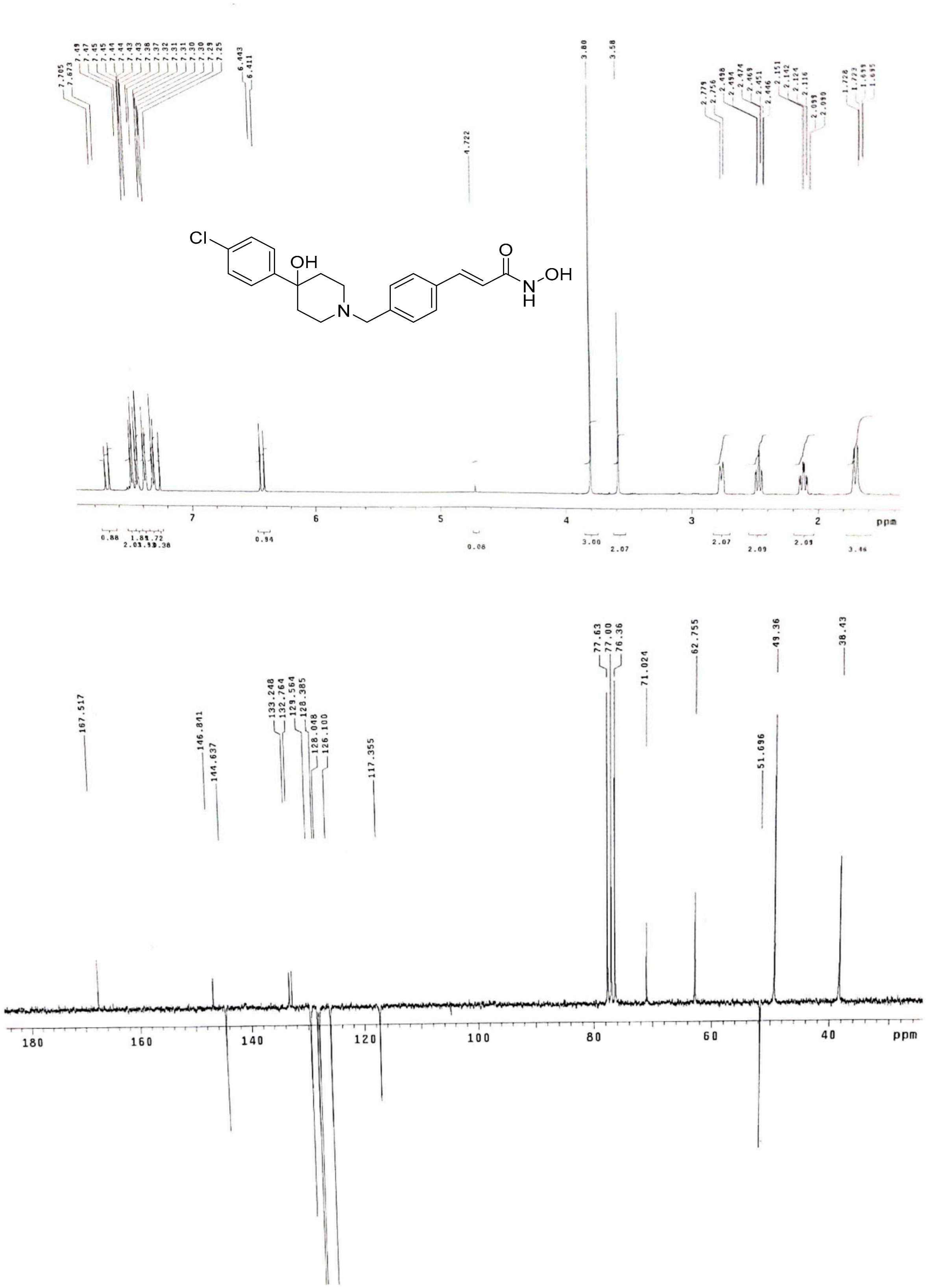

Figure S3. ^{1}H (500 MHz, CD_3OD) and ^{13}C (125 MHz, CD_3OD) (compound **3c**).

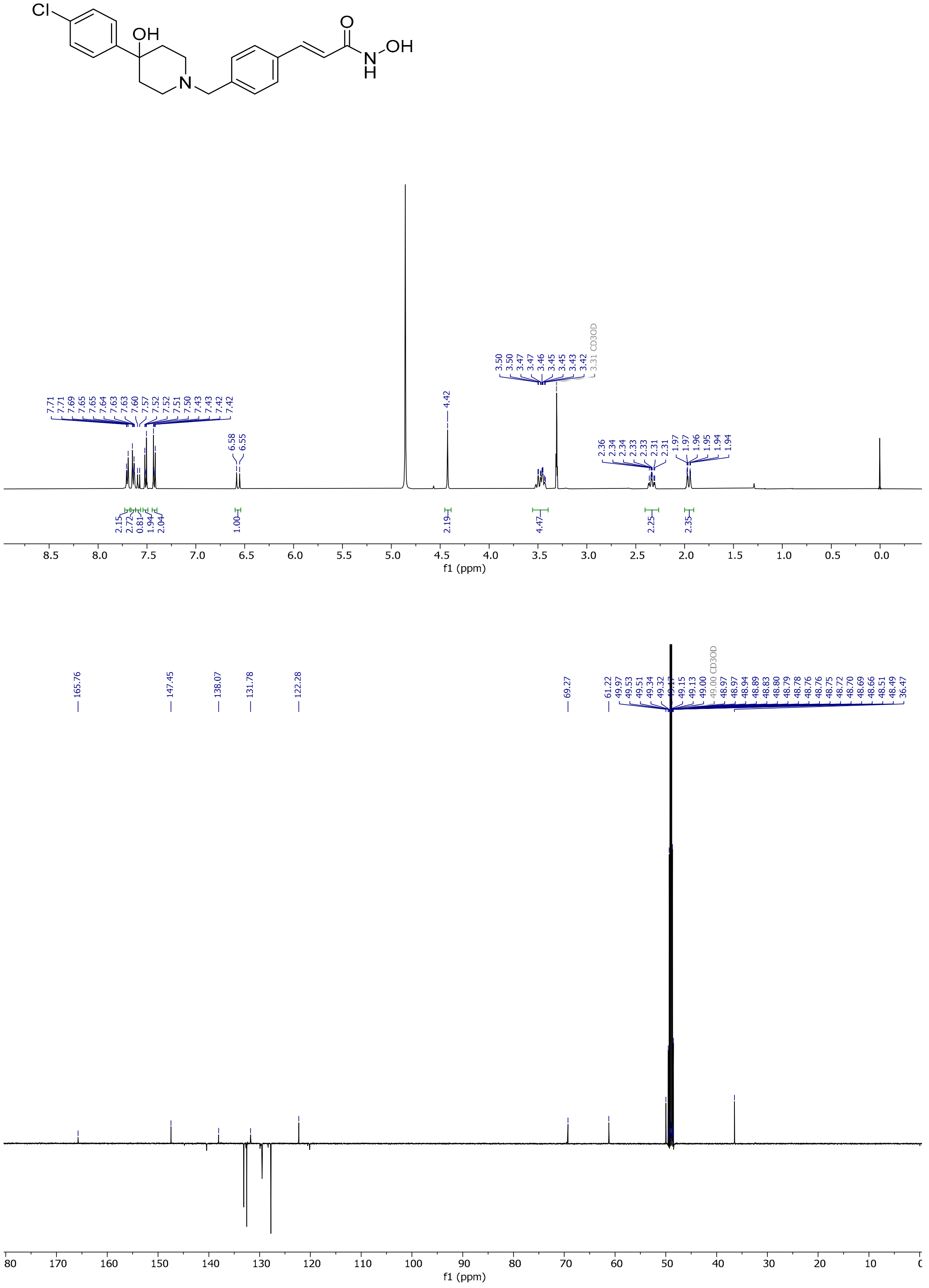

Figure S4. ^{1}H (500 MHz, CD_3OD) and ^{13}C (125 MHz, CD_3OD) (compound **5c**).

HRMS spectra of synthesized compounds

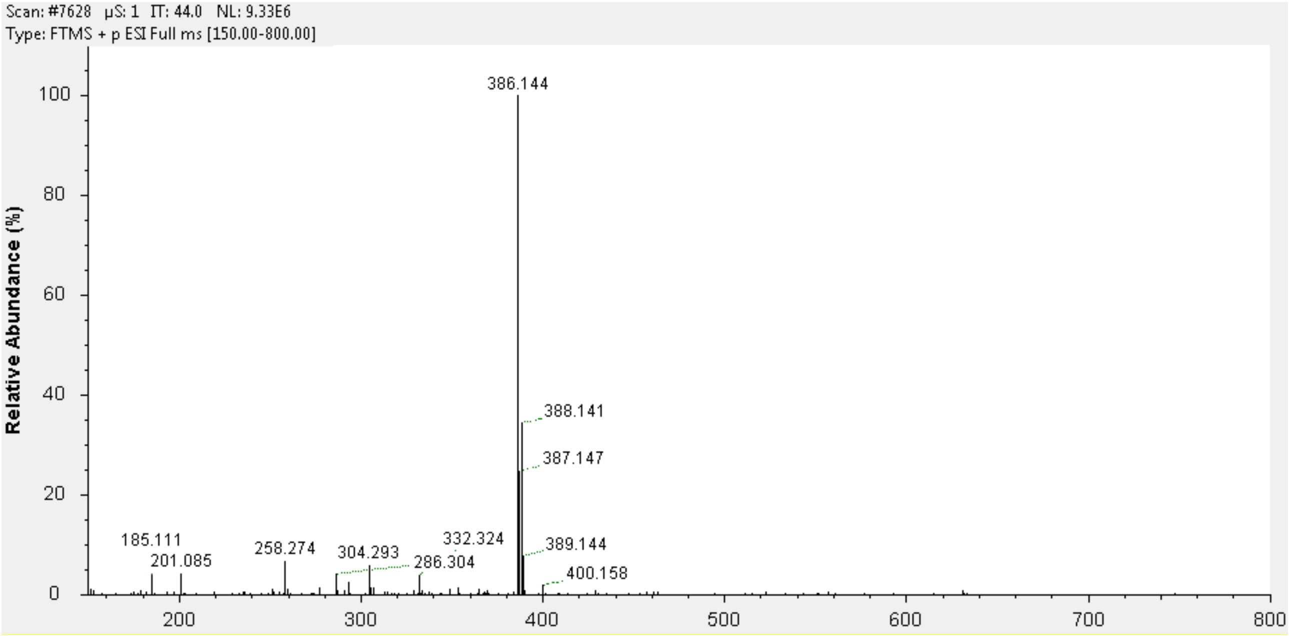

Figure S5. HRMS of compound **3c**. The samples were prepared from pure compounds with a molarity of $1x10^{-6}$ M. Ion optics parameters: sheath gas flow rate, 10 arbitrary units (a.u.); auxiliary gas flow rate, 0 a.u.; spray voltage, 3.50 kV (positive polarity); capillary temperature, 275 °C.

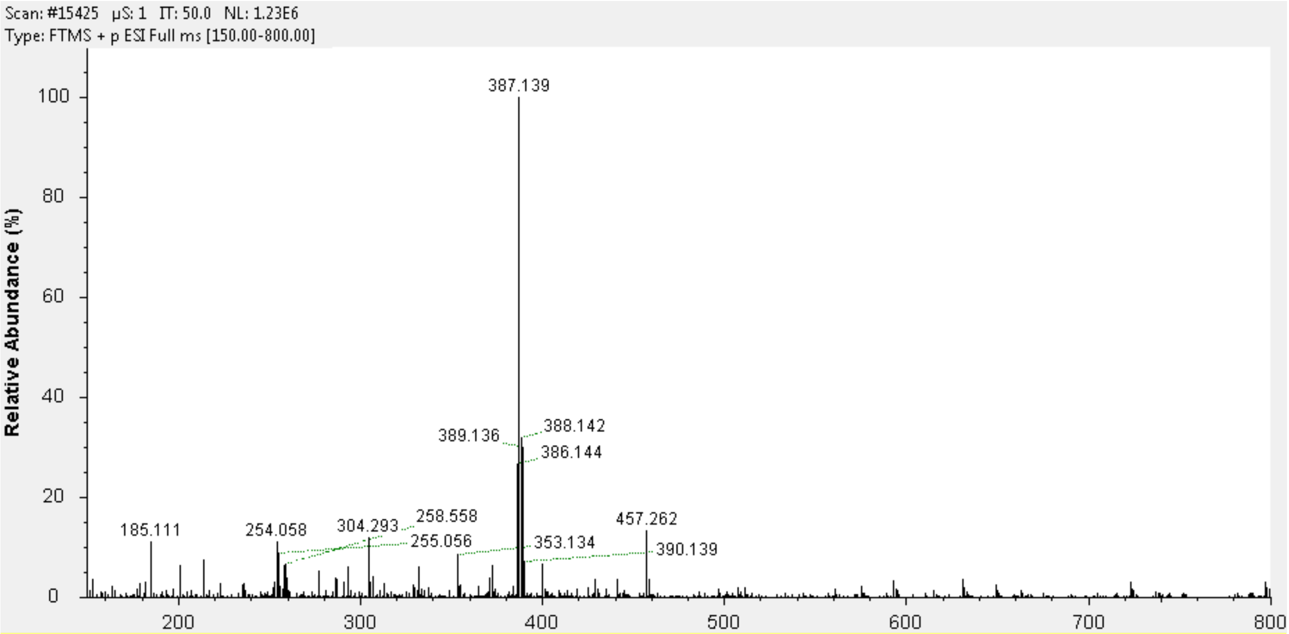

Figure S6. HRMS of compound **5c**. The samples were prepared and processed as described in the legend of Figure S5.
